# A European network for food-borne parasites (Euro-FBP): meeting report on ‘Analytical methods for food-borne parasites in human and veterinary diagnostics and in food matrices’

**DOI:** 10.1186/s13071-017-2506-9

**Published:** 2017-11-09

**Authors:** Christian Klotz, Barbara Šoba, Miha Skvarč, Sarah Gabriël, Lucy J. Robertson

**Affiliations:** 1 Mycotic and Parasitic Agents and Mycobacteria (Unit 16), Department of Infectious Diseases, Robert Koch-Institute, Seestrasse 10, 13353 Berlin, Germany; 2 Institute of Microbiology and Immunology, Faculty of Medicine, University of Ljubljana, Zaloska 4, 1000 Ljubljana, Slovenia; 3 Department of Veterinary Public Health and Food Safety, Faculty of Veterinary Medicine, Ghent University, Salisburylaan 133, 9820 Merelbeke, Belgium; 4 Department of Food Safety and Infection Biology, Faculty of Veterinary Medicine, Norwegian University of Life Sciences, PO Box 8146 Dep., 0033 Oslo, Norway

**Keywords:** Food-borne parasites, Diagnosis, Multi-pathogen detection, Euro-FBP, CYSTINET

## Abstract

Food-borne parasites (FBPs) are a neglected topic in food safety, partly due to a lack of awareness of their importance for public health, especially as symptoms tend not to develop immediately after exposure. In addition, methodological difficulties with both diagnosis in infected patients and detection in food matrices result in under-detection and therefore the potential for underestimation of their burden on our societies. This, in consequence, leads to lower prioritization for basic research, e.g. for development new and more advanced detection methods for different food matrices and diagnostic samples, and thus a vicious circle of neglect and lack of progress is propagated. The COST Action FA1408, A European Network for Foodborne Parasites (Euro-FBP) aims to combat the impact of FBP on public health by facilitating the multidisciplinary cooperation and partnership between groups of researchers and between researchers and stakeholders. The COST Action TD1302, the European Network for cysticercosis/taeniosis, CYSTINET, has a specific focus on *Taenia solium* and *T. saginata*, two neglected FBPs, and aims to advance knowledge and understanding of these zoonotic disease complexes via collaborations in a multidisciplinary scientific network. This report summarizes the results of a meeting within the Euro-FBP consortium entitled ‘Analytical methods for food-borne parasites in human and veterinary diagnostics and in food matrices’ and of the joined Euro-FBP and CYSTINET meeting.

## Background

Food-borne parasites (FBPs), although globally relevant for human and animal health, receive, in general, relatively limited attention in the field of food safety. The reasons for this are manifold, and include a lack of awareness of their actual risk to public health. FBPs are a very diverse group, ranging from various protozoans to all classes of helminth, namely cestodes, nematodes, and trematodes. The biology and transmission cycles of these organisms often differ substantially from each other and, importantly, for many FBP symptoms tend not to develop immediately after infection [1]. In addition, research, diagnostics and detection in food matrices are carried out in specialist laboratories that often focus on only covering a few species or groups of FBP. These factors together lead to the research field being largely fragmented, hampering the visibility of FBPs as important challenges for public health.

In order to foster interdisciplinary exchange of knowledge, in 2015 several European scientists with interests in FBP initiated a network for within the framework of COST (European Cooperation in Science and Technology). This network includes members of over 30 European countries and aims to bring together different players in the FBP field, including, but not limited to, basic researchers, parasitologists and food safety experts [2, 3].

This report highlights the main topics raised during the meeting entitled ‘Analytical methods for food-borne parasites in human and veterinary diagnostics and in food matrices’ that was organized through the auspices of the EU COST Action FA1408 ‘A European Network for Foodborne Parasites: Euro-FBP’ [2, 3], and the joint /co-located meeting of Euro-FBP with the COST Action TD1302, European Network for cysticercosis/taeniosis, CYSTINET [4, 5], which took place in September 2016.

## Overview of the meeting

The meeting was hosted by the Institute of Microbiology and Immunology, Faculty of Medicine, University of Ljubljana and the Slovenian Society for Clinical Microbiology and Hospital Infections of the Slovenian Medical Association and structured as a three-day activity (local organizers: Miha Skvarč and Barbara Šoba).

The first day consisted of mainly internal Action activities, including a formal Management Committee Meeting and individual working group (WG) meetings and a plenary discussion. For further information on the Action’s structure and objectives, please refer to the respective online resources [2, 3]. In addition, two meeting reports were provided on previous activities within the Action: Joke van der Giessen (National Institute for Public Health and the Environment, the Netherlands) presented the outcome of a WG1 activity on ‘Ranking of food-borne parasites in Europe using multicriteria decision analyses’ that was held in February 2016 in Bilthoven, the Netherlands [6]. Rachel Chalmers (*Cryptosporidium* Reference Unit, Public Health Wales Microbiology and Health Protection, Singleton Hospital, UK) reported on a WG2 workshop in Berlin, Germany in June 2016 entitled ‘Towards a consensus on genotyping schemes for surveillance and outbreak investigations of *Cryptosporidium*’ [7].

The second day was organized as a main WG2 activity of the COST action with the central topic ‘Best Practices in Europe regarding detection of FBP’ (see further details below). Invited and submitted presentations, as well as presentations from attendees of ‘short term scientific missions’ (STSM) of year 1 of the Action were given. The aim was to facilitate the knowledge exchange and to strengthen the WG2 Action network.

The third day was a co-located meeting with a related COST Action (TD1302) that comprises a European Network on Taeniosis/Cysticercosis (CYSTINET) [4, 5]. The abstract booklet is also available *via* the Euro-FBP homepage [3] or CYSTINET homepage [4].

## Main meeting Euro-FBP Working Group 2

The second day opened with an introduction and progress reports on the efforts by Christian Klotz (Robert Koch-Institute, Germany) and Michal Slany (Veterinary Research Institute Brno, Czech Republic) to collate a list of European expert laboratories and the methods used by them to detect FBP. The intention is to make the collected data available on the Members’ section of the Action’s home page.

The presentations were sub-structured in six sessions:

### Session 1: *Toxoplasma gondii*

The first session was dedicated to the protozoan parasite *T. gondii*, an apicomplexan parasite that infects all warm-blooded animals and is considered as one of the most important zoonotic parasitoses worldwide, with a high impact on public health.

Jacek Sroka (National Veterinary Research Institute in Pulawy, Poland) gave ‘a brief overview about analytical methods for detection of *T. gondii* in food and water’ and highlighted the importance of sample procedure and concentration steps, given the very different matrices and different parasite stages to be detected (e.g. cysts/bradyzoites in meat, oocysts in water or as contaminants of fresh produce, or tachyzoites in unpasteurized goat milk). It was also highlighted that commercial kits for specific applications are often not available (e.g. detection of oocysts in water, vegetables, etc.) and that bioassays in mice are necessary to determine whether parasites detected in food matrices are infectious.

Vasile Cozma (University of Agricultural Sciences and Veterinary Medicine of Cluj-Napoca, Romania) reported on ‘Serological epidemiology and genotypes of *T. gondii* infections in Romania’. In particular, the successful isolation and genotyping of *T. gondii* (one from human and two from goat kids; all three found to belong to genotype II) were described. Further activities of the consortium in the framework of the European Food Safety Authority (EFSA) funded research activity entitled ‘*T. gondii* in the main livestock species in Europe’ were presented by Joke van der Giessen and Gereon Schares (Friedrich-Loeffler-Institute, Germany). The main goal of these studies focused on elucidating the relationship between seroprevalence in the main livestock species and the presence of *T. gondii* (as demonstrated by PCR detection of *T. gondii* DNA) in meat.

The main conclusions are: (i) lack of information on the topic, especially for cattle, horses, goats and turkeys; (ii) a concordance (pigs, small ruminants and chickens) or a lack of concordance (cattle and horses) between detection of antibodies to *T. gondii* and direct detection of the parasite in meat by PCR; and (iii) absence of antibodies does not guarantee that meat is free of *T. gondii*. A detailed overview of this work can also be found elsewhere [8, 9].

Olgica Djurković-Djaković (University of Belgrade, Serbia) discussed the ‘different goals in man, livestock, food and environmental matrices’ for detecting *T. gondii* infection. She highlighted that for human diagnosis, different detection assays are used depending on the underlying question, e.g. the use of PCR for detection of transmission of the parasite to the foetus or the use of serological methods during pregnancy for risk assessment of congenital infections [10]. From a similar perspective, Miha Skvarč (University of Ljubljana, Slovenia) presented the topic ‘How to predict acute infection with *T. gondii* in the first trimester of pregnancy?’, a question that is still unanswered and produces uncertainties for pregnant women and gynaecologists. One of the tools is to use adjusted IgG avidity test ranges since low avidity in the first trimester can also mean infection before conception. Excellent diagnostic accuracy of adjusted low IgG avidity index to predict acute *T. gondii* infection in the first trimester of pregnancy was presented. However, it was recommended that women should be monitored more often to provide a better clinical and psychological support. The session closed with a presentation from Aleksandra Uzelac (University of Belgrade, Serbia) entitled ‘Diagnosing *T. gondii* infection in haematopoietic stem cell transplant and solid organ transplant patients’. Reliable diagnosis in these patients can be achieved by real-time PCR to detect *T. gondii* in whole blood samples [11]. However, challenges for achieving adequate detection limits were discussed, such as the possible role of PCR-inhibitors or the fact that presence of the parasite in the blood is usually short-lived. She finally discussed whether revision of the diagnostic criteria is necessary.

### Session 2: Invited speaker presentations

The growing availability of genomic datasets and increasing possibilities to analyze data, even for less frequently encountered pathogens, which includes many of the food-borne parasites (FBP), prompted organizers to invite Jessica Kissinger (Institute of Bioinformatics, University of Georgia, USA) to provide a presentation on ‘The value of integrated data for both basic and epidemiological studies: EuPathDB’. She gave an overview of the increasing junction between epidemiology and surveillance on the one side and host-pathogen basic biology on the other side due to next generation sequencing and other -omics technologies. In particular, it was highlighted that the eukaryotic pathogens database (EuPathDB) includes databases of several important FBPs, such as *Entamoeba*, *Cryptosporidium*, *Giardia*, *Toxoplasma* and *Trypanosoma cruzi* [12].

### Sessions 3 and 4: Protozoa

In session 3, two examples were presented on how to adopt new sequencing technologies for relevant epidemiological questions on FBPs. Karin Troell (National Veterinary Institute, Sweden) reported on a successful application of single-cell genomics in *Cryptosporidium* samples derived from cattle. Sequencing of 10 single oocysts from each sample provided acceptable genome data, with coverage varying between 67% and 96% [13]. Future application of this method to assess diversity (e.g. virulence, host specificity, resistance) and recombination events was discussed. Christina Skår Saghaug and Kurt Hanevik (both from the University of Bergen, Norway) reported on a simple bioinformatic workflow to select, extract and analyse *Giardia* genes of interest. This workflow has been applied to ‘analyse single nucleotide variants in 29 different *G. duodenalis* genes responsible for metronidazole metabolism and oxidative stress management’ and the results revealed a higher sequence variation in these genes in *G. duodenalis* Assemblage B isolates than in Assemblage A isolates; the possible relevance for development of resistance was discussed.

Session 4 was mainly dedicated to discussion of evaluation of methods for detection of FBP protozoans in different matrices, such as faecal samples from patients, shellfish, or fruit and vegetables. Here, Rachel Chalmers raised the question ‘Best Practices in Europe: how good are we at diagnosing gastrointestinal food-borne Protozoa?’. An overview of diagnostic tools (molecular methods compared with microscopy methods) to detect *Giardia*, *Cryptosporidium* and *Cyclospora* was given and it was discussed why ‘*Cryptosporidium* spp. and *G. duodenalis* are included in parasitology panels, but *Cyclospora cayetanensis* is rarely included’. She also presented data showing that in faecal parasitological quality control (QC) tests in UK, detection of *Cryptosporidium* tended to be good, but identification of *Cyclospora* was poor [14]. It was concluded ‘that robust algorithms for testing and reporting and maintenance of parasitological competence are needed’.

Another presentation described efforts towards ‘simultaneous detection and characterization of *T. gondii, C. parvum,* and *G. duodenalis* in vegetables and fruits’ by Stéphanie La Carbona (ACTALIA Food Safety Department, France). She presented a workflow that included immunomagnetic separation (IMS) of the parasites followed by detection via qPCR and further analysis by RT-qPCR to estimate viability of the parasites [15]. The workflow and assay performance was overall reported as being positive, but challenges were highlighted based on the ‘variable efficacy depending on foodstuff and parasite’ that must be addressed in future work. In addition, it was clear that the viability assay used did not provide supportive results. A further study to ‘evaluate different protocols for the detection of *Giardia* and *Cryptosporidium* in Mediterranean mussels’ was presented by Panagiota Ligda (Ghent University, Belgium) and her work indicated that pepsin-digestion of samples with subsequent IMS and immunofluorescence antigen test was superior to other techniques using water or ether and no IMS concentration step.

Session 4 was rounded up by Laetitia Kortbeek (National Institute for Public Health and the Environment, the Netherlands) reporting on ‘Risk factors for sporadic cryptosporidiosis cases in the Netherlands: analysis of a three-year population-based case-control study, 2013–2016’. During the three-year study, a species shift from *C. parvum* to *C. hominis* was identified and a correlation between infection and contact with diarrheal cases was deduced from the epidemiological survey. It was concluded that ‘household hand-hygiene improvements could prevent future infections’.

### Session 5: Nematodes

Topics of session 5 were mainly diagnostic measures and investigations of outbreaks with Anisakidae and *Trichinella* spp.

Ivona Mladineo (Institute of Oceanography & Fisheries, Croatia) gave an introductory talk on ‘*Anisakis* sp.: diagnosis from live to canned fish’, highlighting that appropriate molecular tools to identify *Anisakis* to the species level are now available, but that methodology for basic isolation of larvae from fish is still not uniform, thus leading to possible over- or underestimation of actual values of larvae in fish. It was concluded that anisakiasis is ‘still an underestimated and misdiagnosed zoonotic disease in Europe’. Mirosław Różycki (National Veterinary Research Institute, Poland) showed an applied example on ‘Detection of *Anisakis* sp. in fish from the Baltic Sea’, reporting that in their study 14% of herring and 10% of cod were infected with *Anisakis* sp.

*Trichinella* is sometimes still considered as one of the top FBP, although its distribution through Europe is now limited, and it has not been detected in domestic pig herds in many European countries for several decades, and various detection methods for human diagnostics exist. Maria Angeles Gómez-Morales from the European Reference Laboratory for Parasites (EURLP, Istituto Superiore di Sanità, Italy) gave a talk on ‘Serological diagnosis of *Trichinella* infection in humans’, reporting lower sensitivity of commercially available kits (western blot, ELISA) as compared with an EURLP ‘in-house’ assay based on excretory/secretory antigens. She concluded that ‘for *Trichinella* infection, there is an urgent need to harmonize assays among experienced laboratories and compare their performances with the kits present on the market’. The importance of human *Trichinella* diagnostics was also demonstrated by Emília Dvorožňáková (Slovak Academy of Sciences, Slovakia) who presented a summary of ‘Human outbreaks of trichinellosis in Slovakia since year 1980’. Various sources of meat (wild boar, pig, and dog) caused these outbreaks that mainly occurred within endemic areas. In the past 8 years, no further outbreaks were determined possibly due to ‘Slovak and EU legislation that all animals must be examined for the presence of *Trichinella* larvae by digestion method’.

### Session 6: Platyhelminthes (cestodes and trematodes)

In this session, two reports focused on *Echinococcus granulosus* infections, the aetiological agent of cystic echinococcosis (CE), transmitted by embryonated eggs of *E. granulosus* that are shed by infected dogs.

Carmen-Michaela Cretu (Carol Davila University of Medicine and Pharmacy, Romania) reported on ‘Diagnosis and management of echinococcosis - Romanian experience’. Results of various ultrasound screening programmes for CE in Romania were presented that revealed a rate of CE in the rural population of 1.7%. A serological study from Croatia for the presence of *E. granulosus* in patients with cystic liver disease was presented by Mario Sviben (Institute of Public Health, Croatia) and revealed a seroprevalence of 3.9%. The authors concluded that CE is still a health problem in particular regions of Croatia and that there is a clear demand for specific educational, diagnostic and disease management measures. In this context, the European Register of Cystic Echinococcosis (ERCE) was recently initiated in order to create a platform to improve CE related challenges [16]. Laetitia Kortbeek presented a survey in the Netherlands on confirmed *E. multilocularis* cases to test various commercial assays and it was found that ‘for Dutch patients, the specific *E. multilocularis* ELISA and western blot show very low sensitivity and cannot be used for detecting or follow-up of patients with alveolar echinococcosis [17]‘. This implies a necessity for development of alternative detection methods.

Session 6 was completed by an overview presentation of Santiago Mas-Coma (University of Valencia, Spain) on ‘Food-borne trematodiases in Europe’. He categorized human trematode infections in Europe as being either (i) widely distributed (e.g. fascioliasis due to infection with *Fasciola hepatica*), (ii) with a restricted distribution (e.g. opisthorchiasis due to infection with *Opisthorchis felineus*), or (iii) sporadic, rare, or isolated (e.g. dicrocoeliasis due to infection with *Dicrocoelium dendriticum*, heterophyasis due to infection with *Heterophyes heterophyes*, etc.). In summary it was concluded that methods for identification of metacercariae in different foods tend to be still more traditional, being based largely on microscopy, and molecular detection methods are often still at a stage of development.

## Joint meeting: Euro-FBP and Cystinet

Two presenters were invited for the joint meeting. First, Isra Cruz, the Senior Scientific Officer of the Neglected Tropical Diseases Programme, Foundation for Innovative New Diagnostics (FIND), Switzerland, highlighted the activities on ‘Diagnostics development pipeline’ of the non-profit organization FIND [18] that aim to foster the development and implementation of new diagnostic tools for ‘neglected diseases’.

Jessica Kissinger gave the second presentation on ‘What types of information can be gleaned from both whole genome and targeted amplicon NGS data of pathogens?’ and introduced the possibilities of the ‘new’ next generation sequencing technologies in the framework of parasitology.

The third day also included a joint session in which Hélène Yera (Paris Descartes University, France) reported on ‘a real-time PCR assay for the confirmation of neurocysticercosis diagnosis’ that may help to ‘avoid the need for brain biopsy and might be useful for follow-up of both the disease and treatment outcomes’. Teresa Gárate (Instituto de Salud Carlos III, Spain) presented a study on ‘Evaluation of 2B2t recombinant antigen in western blot for diagnosis of cystic echinococcosis’ that may help to improve CE diagnostics in the future. András Laki (Peter Catholic University, Hungary) presented ‘Microfluidic devices for parasitology’ that may, for example, be useful to extract microfilariae from blood samples. A hands-on display of this was provided with the posters. Joke van der Giessen gave a wrap-up presentation of the WG1 activity of Euro-FBP on ‘Ranking food-borne parasites in Europe using multicriteria decision analyses’ [6].

The meeting was then closed by an open discussion on the topic ‘How to envisage (or not) the development/optimization of tools detecting multiple pathogens in the same matrix’. Two discussion groups were formed, followed by a plenary wrap-up session with all participants.

Discussion Group 1 (led by Joke van der Giessen) discussed human and veterinary samples, i.e. blood (whole blood and serum) and stool, whereas Discussion Group 2 (led by Pierre Dorny from the Institute of Tropical Medicine, Belgium) discussed food samples, i.e. meat (meat and meat juice), vegetables and others.

Points (considering FBP) that were addressed within each of the discussion groups and found important to raise in the future, are more focused discussions on the potential need of multi-pathogen detection tools the consideration of their pros and cons, and the question of which pathogens to combine and which detection platform to use.

The need for multi-pathogen detection in food matrices was agreed upon, although the exact combination of which FBPs in which matrices (food matrix and animal/fish spp.) needs to be determined. High throughput systems were envisaged for this multi-pathogen detection within the EU, aiming to combine not only the detection of pathogens, but also other relevant substances such as residues. More classical methods, such as appropriate meat inspection, were advised for more developing regions, where implementation of more complex tools is currently inappropriate for logistical and economic reasons.

Although the importance of multi-pathogen detection in food matrices and surveillance programmes was considered as a logical investment, from a clinical (patient) perspective this is not always obvious. The clinical presentation (signs and symptoms) of human cases would determine the potential pathogens to be explored, and, with regards to animals, the species being investigated would obviously influence which pathogens should be considered. Obviously, the economic factor will also be important in this framework, but simultaneous analyses for several pathogens may, in the long-run and some situations, be of greater value than sequential analyses.

Whether genotyping and WGS was needed, for example in investigating outbreaks and source attribution studies, was a matter of debate between the two groups.

## Conclusion

This meeting addressed a broad topic that is of considerable concern with regard to FBP - their diagnosis in people and animals, and methods of analysis of food matrices for their occurrence. The meeting highlighted, again, the diversity and the complexity of FBP - characteristics that, in part, contribute to the challenges in their diagnosis and detection. In comparison with other food-borne pathogens, detection tools for parasites clearly lag behind, and some examples of efforts to move things forward demonstrated some of the difficulties that need to be addressed. Despite not providing any definitive answers to the challenges, the forum of the cross-parasite meeting, and in particular, the combined efforts of two COST Action meetings, indicate that progress is being made, and opportunities are available to learn from each other.

## Abbreviations

CE: Cystic echinococcosis
FBP: Food-borne parasites
IMS: Immunomagnetic separation
